# The 6 Minute Walk Test and Performance of Upper Limb in Ambulant Duchenne Muscular Dystrophy Boys

**DOI:** 10.1371/currents.md.a93d9904d57dcb08936f2ea89bca6fe6

**Published:** 2014-10-07

**Authors:** Marika Pane, Elena Stacy Mazzone, Serena Sivo, Lavinia Fanelli, Roberto De Sanctis, Adele D’Amico, Sonia Messina, Roberta Battini, Flaviana Bianco, Marianna Scutifero, Roberta Petillo, Silvia Frosini, Roberta Scalise, Gian Luca Vita, Claudio Bruno, Marina Pedemonte, Tiziana Mongini, Elena Pegoraro, Francesca Brustia, Alice Gardani, Angela Berardinelli, Valentina Lanzillotta, Emanuela Viggiano, Filippo Cavallaro, Maria Sframeli, Luca Bello, Andrea Barp, Fabio Busato, Serena Bonfiglio, Enrica Rolle, Giulia Colia, Annamaria Bonetti, Concetta Palermo, Alessandra Graziano, Grazia D’Angelo, Antonella Pini, Alice Corlatti, Ksenija Gorni, Giovanni Baranello, Laura Antonaci, Enrico Bertini, Luisa Politano, Eugenio Mercuri

**Affiliations:** Department of Paediatric Neurology, Catholic University, Rome, Italy; Department of Paediatric Neurology, Catholic University, Rome, Italy; Department of Paediatric Neurology, Catholic University, Rome, Italy; Department of Paediatric Neurology, Catholic University, Rome, Italy; Department of Paediatric Neurology, Catholic University, Rome, Italy; Department of Neurosciences, Unit of Neuromuscular and Neurodegenerative Disorders, Bambino Gesù Children’s Hospital, Rome, Italy; Department of Neurosciences, Psychiatry and Anaesthesiology, University of Messina, Messina, Italy; Department of Developmental Neuroscience, Stella Maris Institute, Pisa, Italy; Department of Paediatric Neurology, Catholic University, Rome, Italy; Department of Experimental Medicine, Cardiomyology and Medical Genetics, Second University, Napoli, Italy; Department of Experimental Medicine, Cardiomyology and Medical Genetics, Second University, Napoli, Italy; Department of Developmental Neuroscience, Stella Maris Institute, Pisa, Italy; Department of Paediatric Neurology, Catholic University, Rome, Italy; Department of Neurosciences, Psychiatry and Anaesthesiology, University of Messina, Messina, Italy; Neuromuscular Disease Unit, Giannina Gaslini Institute, Genoa, Italy; Neuromuscular Disease Unit, Giannina Gaslini Institute, Genoa, Italy; Neuromuscular Center, San Giovanni Battista Hospital, University of Turin, Turin, Italy; Department of Neurosciences, University of Padua, Padua, Italy; Child Neurology and Psychiatry Unit, “Casimiro Mondino” Foundation, Pavia, Italy; Child Neurology and Psychiatry Unit, “Casimiro Mondino” Foundation, Pavia, Italy; Child Neurology and Psychiatry Unit, “Casimiro Mondino” Foundation, Pavia, Italy; Neuromuscular Center, San Giovanni Battista Hospital, University of Turin, Turin, Italy; Department of Experimental Medicine, Cardiomyology and Medical Genetics, Second University, Napoli, Italy; Department of Neurosciences, Psychiatry and Anaesthesiology, University of Messina, Messina, Italy; Department of Neurosciences, Psychiatry and Anaesthesiology, University of Messina, Messina, Italy; Department of Neurosciences, University of Padua, Padua, Italy; Department of Neurosciences, University of Padua, Padua, Italy; Department of Neurosciences, University of Padua, Padua, Italy; Child Neurology and Psychiatry Unit, Maggiore Hospital, Bologna, Italy; Centro Clinico Nemo, Milan, Italy; Department of Neurosciences, Unit of Neuromuscular and Neurodegenerative Disorders, Bambino Gesù Children’s Hospital, Rome, Italy; Department of Neurosciences, Unit of Neuromuscular and Neurodegenerative Disorders, Bambino Gesù Children’s Hospital, Rome, Italy; Department of Paediatric Neurology, Catholic University, Rome, Italy; Department of Paediatric Neurology, Catholic University, Rome, Italy; IRCCS Eugenio Medea, Bosisio Parini, Italy; Child Neurology and Psychiatry Unit, Maggiore Hospital, Bologna, Italy; Developmental Neurology Unit, Neurological InstituteCarlo Besta, Milan, Italy; Centro Clinico Nemo, Milan, Italy; Developmental Neurology Unit, Neurological InstituteCarlo Besta, Milan, Italy; Pediatric Neurology and Psychiatry Unit, Sapienza University, Rome, Italy; Department of Neurosciences, Unit of Neuromuscular and Neurodegenerative Disorders, Bambino Gesù Children’s Hospital, Rome, Italy; Department of Experimental Medicine, Cardiomyology and Medical Genetics, Second University, Napoli, Italy; Department of Paediatric Neurology, Catholic University, Rome, Italy

## Abstract

The Performance of Upper Limb (PUL) test was specifically developed for the assessment of upper limbs in Duchenne muscular dystrophy (DMD). The first published data have shown that early signs of involvement can also be found in ambulant DMD boys. The aim of this longitudinal Italian multicentric study was to evaluate the correlation between the 6 Minute Walk Test (6MWT) and the PUL in ambulant DMD boys. Both 6MWT and PUL were administered to 164 ambulant DMD boys of age between 5.0 and 16.17 years (mean 8.82). The 6 minute walk distance (6MWD) ranged between 118 and 557 (mean: 376.38, SD: 90.59). The PUL total scores ranged between 52 and 74 (mean: 70.74, SD: 4.66). The correlation between the two measures was 0.499. The scores on the PUL largely reflect the overall impairment observed on the 6MWT but the correlation was not linear. The use of the PUL appeared to be less relevant in the very strong patients with 6MWD above 400 meters, who, with few exceptions had near full scores. In patients with lower 6MWD the severity of upper limb involvement was more variable and could not always be predicted by the 6MWD value or by the use of steroids. Our results confirm that upper limb involvement can already be found in DMD boys even in the ambulant phase.

## Introduction

The 6-minute walk test (6MWT) has been recently chosen as the primary outcome measure in several international multicentric clinical trials in Duchenne muscular dystrophy (DMD). Its reliability in a multicentric setting and natural history data have recently been reported by several groups^1^^,^ 2 ^,^ 3 ^,^ 4 ^,^ 5 ^,^ 6 ^,^ 7 ^,^ 8 . In the last few years there has been increasing attention to the assessment of upper limb function^9^ , as it is not captured by the most commonly used motor functional scales, such as the North Star Ambulatory Assessment (NSAA), that mainly focuses on gross motor activities. The Performance of Upper Limb (PUL) is a measure of upper limb function specifically developed for DMD^10^ . The PUL provides both a total score and subscores for the three domains (shoulder, middle and distal) that in DMD are progressively involved with a proximal to distal gradient.

The first published data have shown that the PUL is suitable for multicentric studies, is reliable and can cover the whole spectrum of activities from early signs of proximal involvement in young ambulant DMD boys to distal activities in much older non ambulant DMD boys and adults^11^ . Although as part of the natural progression of DMD, upper limb weakness generally occurs at a later stage compared to lower limb involvement, initial signs of upper limb involvement were already detected in ambulant DMD boys^11^ . These preliminary findings suggested that the scale is ideal for non ambulant boys and adults who have lost ambulation and who are therefore unable to perform the 6MWT or the NSAA, but could also be used as an additional measure in clinical trials in ambulant DMD. Using the PUL in ambulant boys would not only provide additional information on the involvement of upper limbs but could also allow to follow boys who may lose ambulation during the trial and who could therefore not be followed using the 6MWT or the NSAA. Little is known of the age when initial signs of upper limb involvement occur and of the possible correlation with walking abilities.

The aim of this longitudinal Italian multicentric study was to evaluate the correlation between the 6MWT and the PUL in ambulant DMD boys.

## Methods

The study is part of a longitudinal multicentric cohort project involving 11 tertiary neuromuscular centers in Italy aimed at assessing outcome measures in DMD. As part of this overall project, DMD boys and young adults have been prospectively assessed using the 6MWT^5^^,^ 6 ^,^ 7 ^,^ 8 and, in a parallel study performed in the last two years, the PUL^11^ . In the present study we retrospectively evaluated data obtained in the all the ambulant DMD boys from the previous studies who had both PUL and 6MWT.

Patients were recruited between September 2012 and March 2014.

Patient inclusion criteria at baseline were: genetically proven DMD diagnosis, patient still ambulant and able to walk independently for at least 75 meters, no severe or moderate learning difficulties or behavioral problems. Genetic and treatment information were collected and classified following the criteria used in our previous study^7^ .All patients attending the 11 participating centers who fulfilled the inclusion criteria were enrolled in the study.

Steroid use and regimen were also noted.

Details of the training for the physiotherapists involved in the study and of the interobserver reliability for PUL and 6MWT among the centers have already been reported^7^^,^ 8 ^,^ 11 ^,^ 12 .

The data in this study were collected as part of previous published studies on 6MWT and PUL approved by the Ethical Committee of each center and for which informed written consent was obtained. All clinical investigations were conducted according to the principles expressed in the Declaration of Helsinki.

The study was approved by the Ethical Committee of each center.

6MWT

6MWT was performed in all DMD ambulant boys older than 5 according to the ATS guidelines^13^ , modified by having two examiners, one recording time and distances and one staying close to the patient for safety issues.

PUL

The PUL includes 22 items with an entry item to define the starting functional level, and 21 items subdivided into shoulder level (4 items), middle level (9 items) and distal level (8 items) dimension^10^ . For weaker patients a low score on the entry item means high level items do not need to be performed. Scoring options vary across the scale between 0-1 to 0-6 according to performance. Each dimension can be scored separately with a maximum score of 16 for the shoulder level, 34 for the middle level, and 24 for the distal level^11^ . A total score can be achieved by adding the three level scores (max total score 74).

Although this was not a prospective study and there was no design of the timing of the test, as part of our routine 6MWT is always performed at the end of the rest of the clinical assessment , after the boys have a break.

Summary statistics (N, mean, SD, Range) were used. Correlations were evaluated by the Spearman rank correlation coefficients.

## Results

One hundred and sixty-four boys fulfilled the inclusion criteria performing both measures. Their age ranged between 5.0 and 16.17 (mean 8.82, SD 2.42). One hundred and forty-eight were on steroids and 16 were not.

6MWT

The 6minute walk distance (6MWD) ranged between 118 and 557 (mean: 376.38, SD: 90.59).

PUL 

The total scores ranged between 52 and 74 (mean: 70.74, SD: 4.66).

The shoulder domain subscore ranged between 2 and 16 (mean: 13.98, SD: 3.04). The middle domain subscore ranged between 27 and 34 (mean: 33.43, SD: 1.10). The distal domain subscore ranged between 19 and 24 (mean: 23.60, SD: 0.87).

The correlation between the two measures was 0.499. Figure 1 shows the correlation between 6MWD and PUL total scores in the individual boys. Boys with 6MWD above 400 had, with few exceptions scores>70. Between 300 and 400 the scores were more variable and those below 300 meters rarely had full scores.

**Correlation of 6MWT (in meters) and PUL total scores and subscores in the three main domains.**
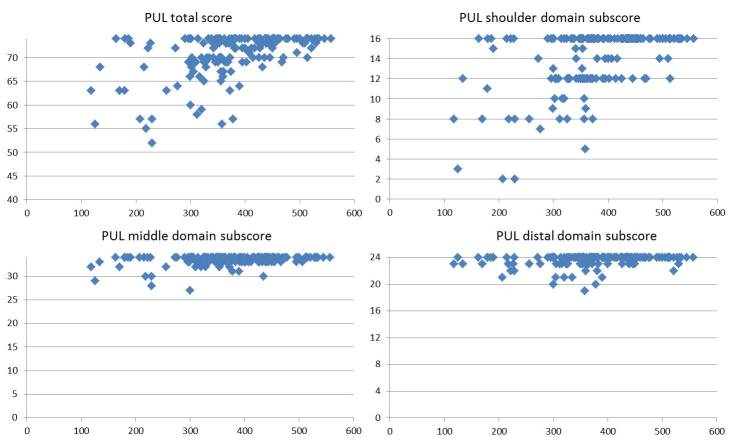

## Discussion

The results of this study confirm that involvement of upper limb function can already be found in ambulant DMD boys^11^ . Not surprisingly the first signs of involvement are related to the shoulder domain with a % of patients not able to perform antigravity shoulder abduction and flexion movements, such as bringing the hand or progressive weights at shoulder or eye level by the side or in front. Because of the proximal to distal gradient of progression of upper limb involvement we observed less changes in the elbow or distal domain that are classically more involved after boys lose ambulation.

When we correlated the PUL with the 6MWT, we found that the correlation was not linear as patients at the stronger end of the spectrum on the 6MWT tended to plateau with full or near full PUL total scores (70 to 74 out of 74). Over 95% of the boys who were able to walk over 400 meters on the 6MWT had total scores between 70 and 74 with over 85 % having scores >72. None of them had scores below 12/16 in the shoulder domain and, with few exceptions they all had full scores on middle and distal domains. These finding confirm that the value of 400 meters, that has recently been used in clinical trials as a cutoff point to identify patients who are unlikely to deteriorate in a short time span, can also identify DMD boys who are at the strong end of the spectrum in regard to upper limb function. In these patients the PUL has a more limited value but its use in longitudinal studies can help to establish the trajectories of changes over time.

Boys with 6MWD between 300 and 400 meters showed a wider range of PUL total scores and not necessarily correlated with the decreasing distance on the 6MWT. Approximately 40% of them had PUL total scores < 70, mainly due to involvement of the shoulder domain. In a few cases, the scores were lower than 60, showing that even in this relatively good range of 6MWT some boys already had early signs of middle and distal involvement. Although we did not aim to systematically assess the effect of steroids on PUL as this will be more accurately assessed in an ongoing longitudinal study, we checked if the variability in the PUL scores could be justified by possible differences in steroid treatment. This was not the case as all the patients with PUL total scores below 60 were on steroids and more than half of them on a daily regime.

Not surprisingly, the number of boys with full or near full PUL total scores in the group with 6MWT below 300 meters was much lower. The lower total scores reflected a higher number of boys who did not have full scores both in the middle and distal domain.

## Conclusions

In conclusion, our results confirm that upper limb involvement can already be found in DMD boys even in the ambulant phase. The scores on the PUL largely reflect the overall impairment observed on the 6MWT but the correlation was not linear. The use of the PUL appears to be less relevant in the very strong patients with 6MWT above 400 meters, that are often excluded from clinical trials. In patients with lower 6MWT early signs of involvement are not only limited to shoulder involvement but also, in some cases, to the loss of the ability to fully perform some activities in the middle and distal domain. The severity of upper limb involvement was variable and could not always be predicted by the overall weakness and fatigability measured by the 6MWT or by the use of steroids. The concomitant use of the PUL appears to be even more relevant in the patients with 6MWT results below 350 meters, and especially in those below 230 meters who are more likely to lose ambulation within two years^6^ . In these boys the PUL will provide the possibility to monitor possible functional changes also during and after loss of ambulation. Longitudinal studies are in process in both ambulant and non ambulant DMD boys and adults in order to better understand the changes of the PUL scores over time and the possible effect of different variables, such as age and steroids on the progression of upper limb involvement.

## Competing Interests

The authors have declared that no competing interests exist. They report the following disclosures: Dr. Pane, E. Mazzone, Dr. Sivo, Dr. Fanelli, Dr. De Sanctis, Dr. D'Amico, Dr. Messina, Dr. Battini, Dr. Bianco, Dr. Scutifero, Dr. Petillo, Dr. Frosini, Dr. Scalise, Dr. Vita, Dr. Bruno, Dr. Pedemonte report no disclosures. Dr. Mongini has served on a scientific advisory board for Telethon Italy; has received funding for travel from Genzyme Corporation; and has received research support from AIFA (Italian Government Drug Agency) and Telethon Italy. Dr. Pegoraro has served on a scientific advisory board for BioMarin Pharmaceutical Inc.; has received funding for travel from Genzyme Corporation; and has received speaker honoraria from MedaPharma; and receives research support from Wellstone and Telethon Italy. Dr. Brustia, Dr. Gardani, Dr. Berardinelli, Dr. Lanzillotta, Dr. Viggiano, Dr.Cavallaro, Dr. Sframeli, Dr. Bello, Dr. Barp, Dr. Busato, Dr. Bonfiglio, Dr. Rolle, Dr. Colia, Dr. Bonetti, Dr. Palermo, Dr. Graziano, Dr.D’Angelo, Dr. Pini, Dr. Corlatti, Dr. Gorni, Dr Baranello , Dr. Antonaci report no disclosures. Dr. Bertini is site PI for the PTC extension study of Ataluren in DMD , for the GSK study on exon skipping. He also receives funds from the Italian Telethon, the Italian Ministry of Health and SMA Europe for observational studies on outcome measures. Dr. Politano reports no disclosure. Dr. Mercuri is site PI for the PTC extension study of Ataluren in DMD , for the GSK Prosensa and Sarepta studies on exon skipping. He also receives funds from the Italian Telethon and SMA Europe. He has acted as advisory board for Acceleron Pharma, Shire and PTC Therapeutics, Inc, Prosensa .

## Authorship Notes

Marika Pane, Elena Stacy Mazzone, and Serena Sivo contribute equally to this manuscript.

Correspondence: *mercuri@rm.unicatt.it (E. Mercuri).*

## References

[1] Henricson E, Abresch R, Han JJ, et al. The 6-minute walk test and person-reported outcomes in boys with duchenne muscular dystrophy and typically developing controls: longitudinal comparisons and clinically-meaningful changes over one year. PLoS currents 2013;5. 10.1371/currents.md.9e17658b007eb79fcd6f723089f79e06 PMC3712467 23867975

[2] McDonald CM, Henricson EK, Abresch RT, et al. The 6-minute walk test and other endpoints in Duchenne muscular dystrophy: Longitudinal natural history observations over 48 weeks from a multicenter study. Muscle Nerve 2013;48:343-356. 10.1002/mus.23902 PMC3824082 23681930

[3] McDonald CM, Henricson EK, Abresch RT, et al. The 6-minute walk test and other clinical endpoints in duchenne muscular dystrophy: Reliability, concurrent validity, and minimal clinically important differences from a multicenter study. Muscle Nerve 2013;48:343-356. 10.1002/mus.23905 PMC3826053 23674289

[4] McDonald CM, Henricson EK, Han JJ, et al. The 6-minute walk test in Duchenne/Becker muscular dystrophy: longitudinal observations. Muscle Nerve 2010;42:966-974. 10.1002/mus.21808 21038378

[5] Pane M, Mazzone ES, Sormani MP, et al. 6 minute walk test in Duchenne MD patients with different mutations: 12 month changes. PLoS One 2014;9:e83400. 10.1371/journal.pone.0083400 PMC3885414 24421885

[6] Mazzone ES, Pane M, Sormani MP, et al. 24 month longitudinal data in ambulant boys with Duchenne muscular dystrophy. PLoS One 2013;8:e52512. 10.1371/journal.pone.0052512 PMC3543414 23326337

[7] Mazzone E, Vasco G, Sormani MP, et al. Functional changes in Duchenne muscular dystrophy: a 12-month longitudinal cohort study. Neurology 2011;77:250-256. 10.1212/WNL.0b013e318225ab2e 21734183

[8] Mazzone E, Martinelli D, Berardinelli A, et al. North Star Ambulatory Assessment, 6-minute walk test and timed items in ambulant boys with Duchenne muscular dystrophy. Neuromusc Disord 2010;20:712-716. 10.1016/j.nmd.2010.06.014 20634072

[9] Mazzone E VG, Palermo C, Bianco F, Galluccio C, Ricotti V, Castronovo AD, Di Mauro MS, Pane M, Mayhew A, Mercuri E. A critical review of functional assessment tools for upper limbs in Duchenne muscular dystrophy. Dev Med Child Neurol 2012;54:879-85 10.1111/j.1469-8749.2012.04345.x 22713125

[10] Mayhew A, Mazzone ES, Eagle M, et al. Development of the Performance of the Upper Limb module for Duchenne muscular dystrophy. Dev Med Child Neurol 2013;55:1038-1045. 10.1111/dmcn.12213 23902233

[11] Pane M, Mazzone ES, Fanelli L, et al. Reliability of the Performance of Upper Limb assessment in Duchenne muscular dystrophy. Neuromusc Disord 2014;24:201-206. 10.1016/j.nmd.2013.11.014 24440357

[12] Mazzone ES, Messina S, Vasco G, et al. Reliability of the North Star Ambulatory Assessment in a multicentric setting. Neuromusc Disord 2009;19:458-461. 10.1016/j.nmd.2009.06.368 19553120

[13] ATS statement: guidelines for the six-minute walk test. Am J Respir Crit Care Med 2002;166:111-117. 10.1164/ajrccm.166.1.at1102 12091180

